# Is There any Mean to Postpone The Menopausal Ovarian Senescence?

**DOI:** 10.22074/ijfs.2020.5797

**Published:** 2019-11-11

**Authors:** Awad Hegazy Abdelmonem

**Affiliations:** Consultant of Gynecology, Obstetrics and Infertility and Professor and Former Chairman of Anatomy and Embryology Department, Faculty of Medicine, Zagazig University, Zagazig, Egypt

## Abstract

The ovarian reserve (OR) gradually decreases throughout the female fertile life. This continuous depletion in OR is irreversible. This occurs through a programmed cell death, known as apoptosis. Some factors hasten such depletion, such as chemo- and radio-therapy. Others have been investigated in trials to preserve the OR including gonadotropins, cytokines, growth hormones, nitric oxide and reorganization of the actin cytoskeleton. Loss of OR occurs normally at the menopausal age, a stage called menopausal ovarian senescence. At some periods, there are other sources for ovarian hormones that are away from the ovary, like during use of contraceptive pills and at pregnancy after formation of placenta. Future trials to preserve ovarian follicles at these periods might postpone the onset of menopause and hence lengthen the fertile female age.

## Introduction

Ovary is the primary sex organ in females. It is the only abdominal organ that is not covered by peritoneum (1). It contains hundreds of thousands (about 400000) primary follicles at puberty. These follicles support fertile period of female life. At the mid-30s years, there is an increase in the pace of oocyte depletion to reach about 25000 by the age of forty years. This means that the ovarian reserve (OR) decreases gradually till ends at the menopause. This continuous depletion in OR, as an irreversible process, is variable from a woman to the other and depends on many factors such as genetics, age, drugs, irradiations and other environmental variables. For example, hormonal suppression as well as chemo- and radio-therapy might hasten follicle depletion causing deleterious effects on the OR and ovarian function (2). On the other hand, there are environmental factors, including nutrition which might improve condition of ovarian aging (3).

OR which represents the female potentials for fertility could be tested by measuring anti-Mullerian hormone (AMH). Women with low AMH levels should be counseled regarding the option of fertility preservation or tendency to attempt pregnancy at early stage of their fertility life (4). Moreover, low response of ovaries to ovulation induction by gonadotrophins is an indication of reduced OR and frequently associated with occurrence of early menopause (5). 

At each ovarian cycle, some of the follicles begin to enlarge under hormonal control, till one of them reaches the stage of mature Graafian follicle. This ripe follicle approximates the ovarian surface epithelium (OSE) and releases its oocyte into the abdominal “peritoneal” cavity, a process that is known as ovulation (6). At the same time, other follicles degenerate throughout their journey of maturation. Therefore, during each cycle, many follicles “approximately 1000 in terms of quantity” are lost (7).

At pregnancy, functions of ovaries become stationary. By that means, no hormone is produced from ovary after formation of the placenta taking over the role of ovary in production of the hormones supporting pregnancy and enhancing enlargement of breasts and uterus. Removal of ovaries after the first trimester does not affect the progress of pregnancy (7, 8). It has been found that many follicles are enlarged during pregnancy but no ovulation occurs, while it is stopped after fertilization. This might be because of the proliferation of OSE cells and increase in the thickening (9). Hormonal changes that are responsible for prevention of ovulation at pregnancy might be similar to that found with use of contraceptive pills. However, Cooper and Adigun (10) stated that the progesterone is the main hormone in the pills responsible for prevention of ovulation, by inhibiting follicle development.

Menopause is a females’ stage of life representing about one third of their lives. Such stage is characterized by depletion of ovarian follicles and hence cessation of menstruation and loss of fertility. There is a loss of cyclic production of steroid hormones. The most noticed hormonal change corresponding to female reproductive aging in menopause is the subtle rise of follicle stimulating hormone (FSH) (1). 

Degeneration of follicles or their atresia occurs through a programmed cell death, known as apoptosis (11). Several agents have been tested to rescue these cells from apoptosis. These include gonadotropins, estrogens, cytokines, growth hormones, nitric oxide and reorganization of the actin cytoskeleton (11-13). Wu et al. (14) noticed that use of resveratrol, which is a natural plant derivative, in mice could reduce cell apoptosis of oogonia induced by chemotherapy. They suggested that resveratrol might be used as a potential therapeutic drug against induced ovarian aging. Although such trials are in their infancy, our hope is enlarging to find a mean to preserve the ovarian follicles in vivo or to slow the natural rate of atretic loss, especially when two ovaries are in a state of stagnation such as occurring at pregnancy after the first trimester as well as in cases of use of contraceptive pills. This could lengthens the fertile female age and postpone the menopausal onset for additional years. 
